# Mortality associated with avian reovirus infection in a free-living magpie (*Pica pica*) in Great Britain

**DOI:** 10.1186/s12917-015-0329-5

**Published:** 2015-02-07

**Authors:** Becki Lawson, Akbar Dastjerdi, Sonal Shah, David Everest, Alejandro Núñez, Ann Pocknell, Daniel Hicks, Daniel L Horton, Andrew A Cunningham, Richard M Irvine

**Affiliations:** Institute of Zoology, Zoological Society of London, Regent’s Park, London, NW1 4RY UK; Animal and Plant Health Agency (APHA), Weybridge, New Haw, Addlestone, Surrey, KT15 3NB UK; Finn Pathologists, One Eyed Lane, Weybread, Diss, Norfolk, IP21 5TT UK; School of Veterinary Medicine and Science, University of Nottingham, Sutton Bonington Campus, Leicestershire, LE12 5RD UK; School of Veterinary Medicine, University of Surrey, Guildford, Surrey GU2 7XH UK

**Keywords:** Avian reovirus, Magpie, *Pica pica*, Hepatic necrosis, Splenic necrosis

## Abstract

**Background:**

Avian reoviruses (ARVs) cause a range of disease presentations in domestic, captive and free-living bird species. ARVs have been reported as a cause of significant disease and mortality in free-living corvid species in North America and continental Europe. Until this report, there have been no confirmed cases of ARV-associated disease in British wild birds.

**Case presentation:**

Sporadic individual magpie (*Pica pica*) mortality was detected at a single site in Buckinghamshire, England, April-September 2013. An adult female magpie was found moribund and subsequently died. Post-mortem examination identified hepatomegaly and splenomegaly as the most severe macroscopic abnormalities. Histopathological examination revealed extensive hepatic and splenic necrosis. Transmission electron microscopy (TEM) identified virions of a size (circa 78 nm diameter) and morphology consistent with ARV in both the liver and the small intestinal (SI) contents. Nucleic acid extracted from pooled liver and spleen was positive on both a pan-reovirus nested PCR targeting the RNA-dependent RNA polymerase gene and a PCR using primers specific to the ARV sigma C protein gene. Virus isolated from the liver and the SI contents was characterised by a syncytial-type cytopathic effect, a reovirus-like appearance on TEM and sequence identical to that from PCR of tissues. *In situ* hybridisation confirmed co-localisation of ARV with lesions in the liver and spleen, implicating ARV as the causative agent. Splenic lymphoid atrophy and necrotic stomatitis associated with *Aspergillus fumigatus* infection were consistent with generalised immunosuppression and resultant opportunistic infection.

**Conclusions:**

The pathology and comprehensive virus investigations in this case indicate ARV as the primary pathogen in this magpie, with concurrent secondary infection subsequent to immunosuppression, as has been observed with reoviral infections in other bird species. ARV should be considered as a differential diagnosis for magpie, and potentially other corvid, disease and mortality incidents. This is the first demonstration of ARV-associated mortality in a wild bird in Britain. The prevalence and significance of ARV infection in British wild birds, and its implications for poultry and captive bird health, are currently unknown.

## Background

Avian reoviruses (ARVs) comprise a diverse genus of non-enveloped, double-stranded RNA viruses with a segmented genome within the family Reoviridae [1]. ARVs are associated with a range of disease presentations in birds from various orders. In chickens (and to a lesser extent in turkeys), arthritis/tenosynovitis is the most common disease presentation caused by ARVs [2,3]. Myocarditis, pancreatitis, hepatitis and enteric syndromes have also been reported in Galliformes [4-7]. Necrotising hepatitis and splenitis are typically reported in psittacine and anseriform species with ARV infection [8,9].

Reoviruses have been isolated from both healthy and diseased birds and are frequently diagnosed with concurrent infections; careful evaluation is required to determine their clinical significance in each case. Faeco-oral transmission is considered to be the primary route of transmission [1].

ARV has been reported as a cause of significant disease and/or mortality in free-living corvid species in North America [(American crow, *Corvus brachyrhynchos*) in the USA, 2001 [10] and Canada, 2004 [11]] and Europe [(hooded crow, *Corvus corone cornix*) in Finland, 2002 [12] and carrion crow (*Corvus corone*) in Belgium, 2004 [13]]. To date, there are no reports of ARV infection associated with clinical disease in British wild birds.

Here, we present a case of ARV infection in a wild magpie (*Pica pica*) from southern England, associated with hepatic and splenic necrosis, where the pathological findings indicate the reovirus infection is likely to be the primary and significant disease contributing to the cause of death.

## Case presentation

### Incident history

A single magpie was observed with non-specific signs of malaise in a rural garden in Buckinghamshire, England, in April 2013. The bird was in a moribund condition and died soon after capture. Two other magpies, also with lethargy and non-specific malaise, were subsequently observed at the same site, one in May 2013 and the second in September 2013. The observed development of disease in these birds was similar, with progressive weakness developing over an approximately two week period. The first bird was found dead. The latter two were presumed to have died, but their carcases were not recovered for further investigation, illustrating one of the challenges of wildlife disease surveillance [14].

### Post-mortem examination and ancillary diagnostic testing

**Figure 1:**
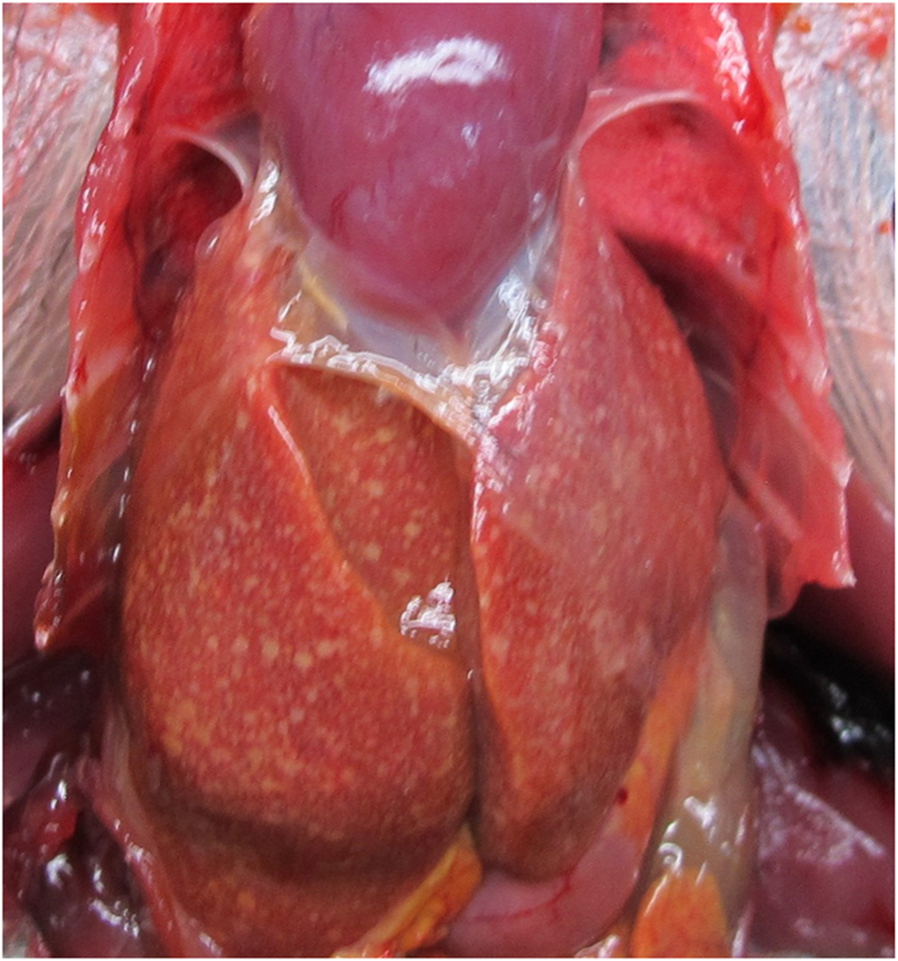
**Macroscopic appearance of the liver.** The liver at gross post-mortem examination showing enlargement, orange discolouration of the parenchyma and miliary distribution of pale tan foci.

Pathological examination of the recovered carcase (the first case seen) was performed using a standardised protocol comprising systematic external and internal examination of body systems, as described by [15]. This confirmed it was an adult female magpie in emaciated body condition with wasted pectoral muscle mass and absence of body fat. The carcase had not been frozen and was in a good state of preservation (the interval from the carcase being found to examination was circa 72 hours). The skin was tightly adherent to the subcutis, suggestive of generalised dehydration. The wing and tail plumage were in poor condition with fractured vane tips and stress marks of moderate severity, consistent with chronic poor health. Macroscopic examination revealed severe hepatomegaly with markedly swollen liver lobes, orange parenchymal discolouration with pale tan-coloured miliary stippling of the hepatic parenchyma (Figure 1). Concurrent splenomegaly was present with diffuse parenchymal congestion and haemorrhage. No evidence of the bursa of Fabricius was observed. Bilateral lung congestion was grossly visible. The air sacs and pericardium had pale orange discolouration, with mild thickening and a cloudy appearance.

Generalised, severe necrotic stomatitis was present with cream-coloured, crumbly debris adherent to the palate and floor of the mouth extending around the base of the tongue. The proventriculus and gizzard were empty. The small intestine contained a moderate volume of brown fluid content with some blood-staining, whilst the small intestinal (SI) serosal vessels appeared grossly congested. The large intestine contained a moderate volume of paste-like dark brown-coloured contents.

The adrenal glands were both prominent, possibly enlarged. Congestion of the cerebral vasculature was noted on gross inspection. No other significant macroscopic abnormalities were detected.

Bacteriological examination of liver, SI contents and the oral lesion was performed, including incubation under aerobic, anaerobic and microaerophilic conditions, following the protocols described by [15]. Confluent near pure growths of *Escherichia coli* were recovered from the liver and SI contents with no other significant organisms isolated. Anaerobic cultures of the liver and SI contents were negative for *Clostridium* spp. A liver impression smear with Ziehl-Neelsen staining was negative for acid-fast bacilli.

*Aspergillus fumigatus* was isolated and identified on the basis of colony and conidial morphology following culture from the oral lesion using Sabouraud’s Dextrose agar with chloramphenicol (Oxoid, UK; Reference P00358A) at 25°C.

Direct microscopical examination of a saline mount crush preparation of the oral lesions was negative for protozoan or metazoan parasites. A circa 5 mm^3^ section of oral lesion was inoculated into Trichomonas media 2 (Oxoid, UK; Reference LR0027A) and incubated at 30°C in an aerobic incubator for 5 days; media was observed for trichomonad parasites at 2, 3 and 5 days as per the protocol described by [15] and was negative for *T. gallinae*. There was no evidence of parasites on direct microscopical examination of the SI contents.

**Figure 2:**
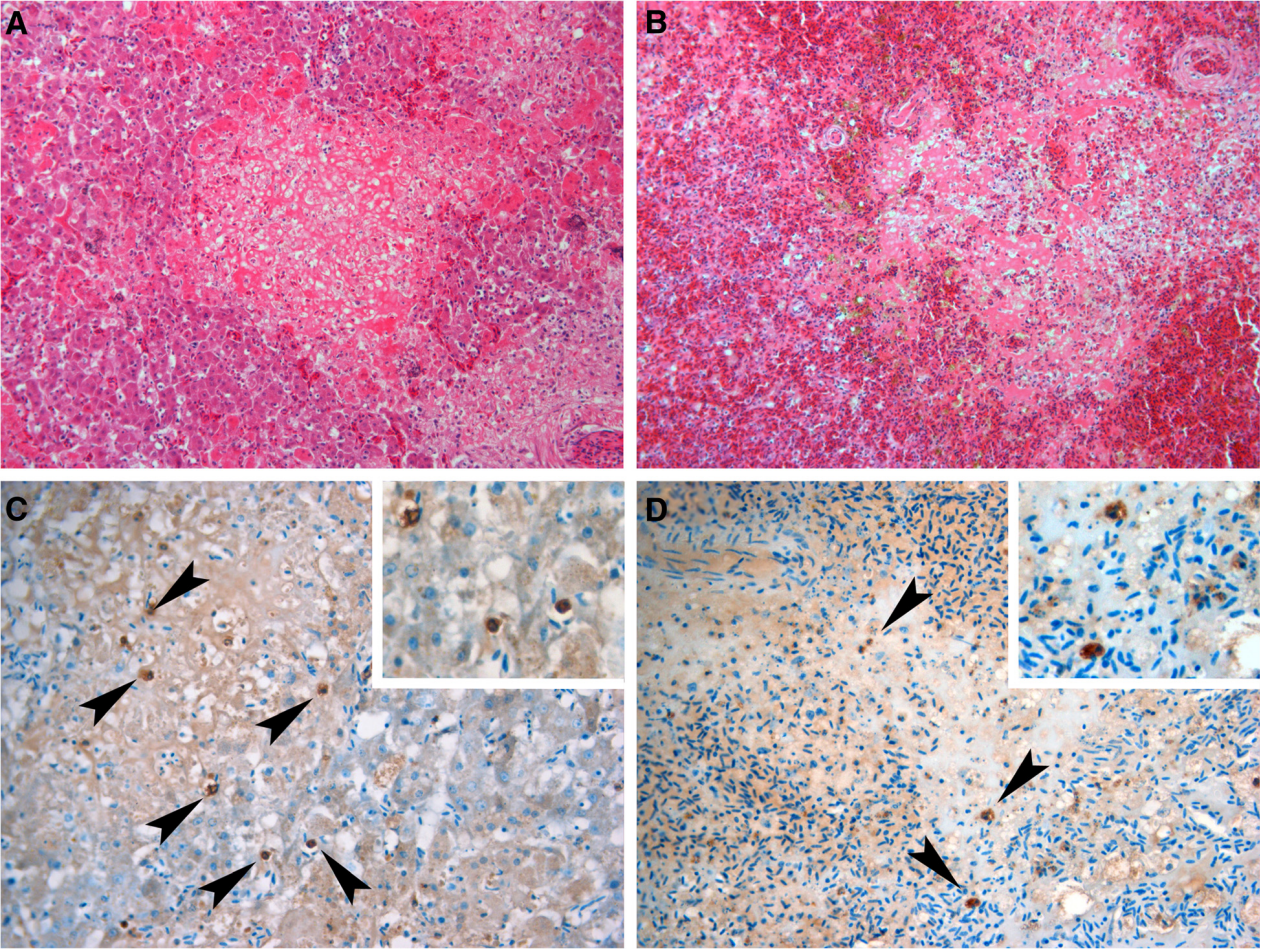
**Histopathology and in-situ hybridisation. A**. Discrete focal to coalescent acute hepatic necrosis. H&E. 200x. **B**. Fibrinous splenic necrosis. H&E. 200×. **C**-**D**. Demonstration of ARV RNA by in-situ hybridisation in intralesional cells (arrowheads) in liver **(C)** and spleen **(D)**. 400x.

A range of tissue samples was fixed in 10% neutral-buffered formalin and prepared for histopathological examination using standard techniques. In addition to the standard H&E preparation, sections were examined with special stains: Giemsa, Gram-Twort, Perls’ Prussian Blue, Periodic Acid-Schiff, and Ziehl-Neelsen. Histopathological examination identified coalescing, submassive areas of acute hepatic necrosis, associated with haemosiderin deposition (identified using the Perls’ stain) and considered sufficient to account for death due to hepatic failure (Figure 2A). Mild, focal cholangitis with bile duct ectasia was noted in association with luminal trematodes. Focally extensive, acute, fibrinous splenic necrosis was seen, with severe congestion and focal haemorrhages throughout the parenchyma (Figure 2B). Diffuse lymphoid atrophy was also present in the spleen. Diffuse adrenocortical hyperplasia was noted. No lesions were detected in the sections of brain examined histologically. No significant organisms were detected on examination of sections stained with H&E or any of the special stains used.

### Virological investigations

Nucleic acid was extracted from pooled brain and kidney using TRIzol® Reagent (Life Technologies) following the manufacturer’s protocol, and screened using a pan-flavivirus [16] and West Nile virus specific real-time PCR [17]: both tests were negative. Nucleic acid was extracted from pooled liver and spleen using the EZ1 RNA Universal Tissue Kit (Qiagen) on an EZ1 Advanced XL robot following the manufacturer’s protocol and was screened using a pan-herpes virus PCR [18] which was negative.

Negative contrast TEM was performed on both SI contents and liver homogenate using standard protocols [19]. Virus particles of a size (circa 77–78 nm diameter), shape and surface morphology consistent with reovirus were identified in both samples.

A pan-reovirus nested PCR targeting the L2 genome segment [20] was performed on the nucleic acid extracted from pooled liver and spleen. Complementary DNA was generated from 200 ng nucleic acid using SuperScript™ III reverse transcriptase (Life Technologies) at 50°C for 30 minutes as instructed by the manufacturer. Conditions for the PCR were as described [20], but using the Fast Cycling PCR kit (Qiagen). ARV PCR product was purified using the MinElute PCR Purification Kit (Qiagen) and DNA sequencing was performed using BigDye Terminator Ready Reaction Mix (Life Technologies) on an ABI 3130xL genetic analyser platform (Life Technologies). Sequence reads were checked for ambiguities using SeqMan software of the DNASTAR Lasergene Core Suite (DNASTAR, Inc.) and primer sequences were edited out before BLAST search (http://blast.ncbi.nlm.nih.gov/Blast.cgi). An amplicon of the size expected for reovirus L2 gene RNA-dependent RNA polymerase (circa 274 bp) was obtained.

**Figure 3:**
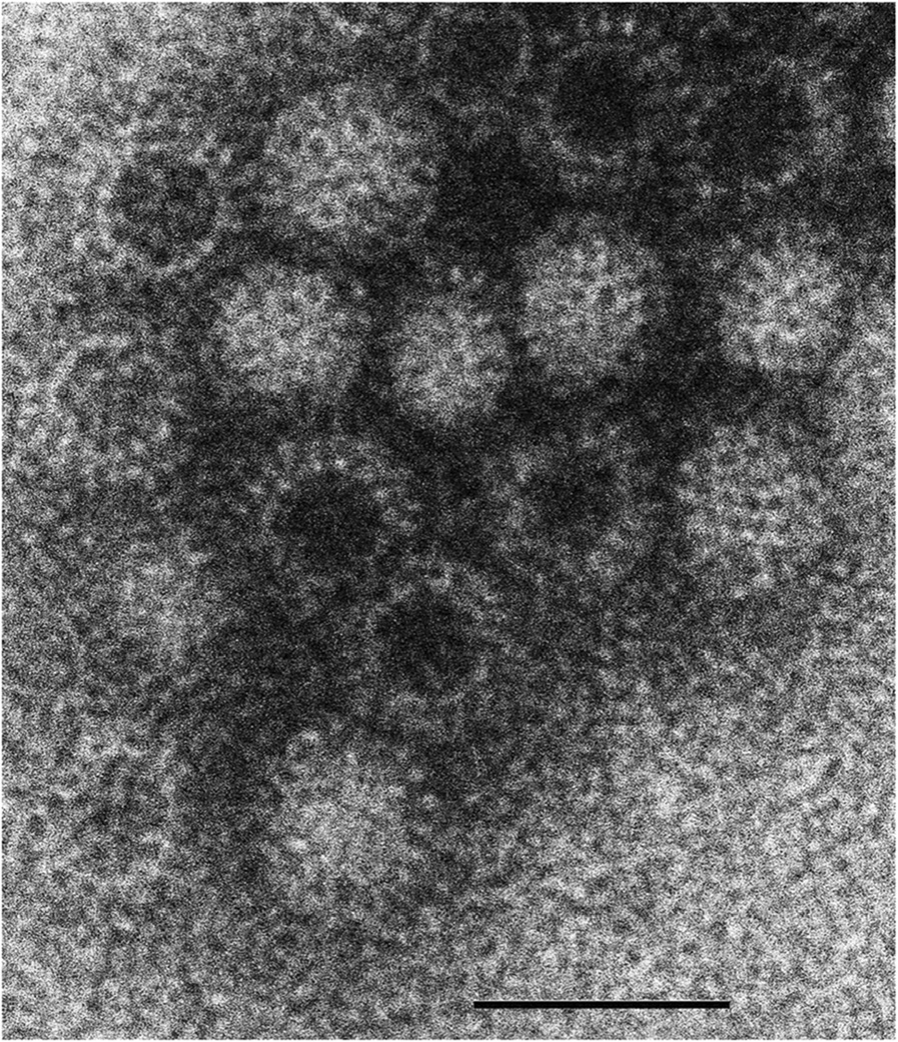
**Transmission electron micrograph of a reovirus detected in the magpie.** Image from magpie liver tissue culture material. *Bar = 100 nm.*

Virus isolation of liver, kidney and SI contents was attempted; a pool of kidney and liver homogenate, and a sample of SI contents were separately used to seed Vero cell line C1008 in the presence of 10% foetal calf serum (FCS) and Dulbecco’s Modified Eagle Medium (Life Technologies). Both the liver/kidney pool and SI contents cultures resulted in a syncytial-type cytopathic effect. TEM on the cell pellet of the fourth tissue culture passage from each culture revealed virus particles (Figure 3) with an indistinguishable appearance to those in the post-mortem tissues.

### In-situ hybridisation

A biotin-labelled probe for the magpie reovirus in-situ hybridisation (ISH) assay was generated using first round pan-reovirus PCR as the template. The labelling PCR mastermix consisted of 5 μl 10x AccuTaq™ LA DNA Polymerase buffer (Sigma-Aldrich), 1 μl of each second round PCR primer (Eurofins, 20 pmol/μl), (1 mM of each dATP, dCTP, dGTP, 0.65 mM dTTP and 0.35 mM biotin-16-dUTP, Roche Applied Science), 0.5 μl AccuTaq™ LA DNA Polymerase (Sigma-Aldrich, 5 u/μl), 28.5 μl water and 5 μl template. The cycling conditions were 95°C for 5 minutes, 40 cycles of 95°C for 15 seconds, 47°C for 20 seconds, 72°C for 30 seconds and 72°C for 2 minutes. Porcine enteric diarrhoea virus (PEDV) nucleic acid was labelled in a similar manner and used as a negative target control. The labelled products were purified using the MinElute PCR purification kit (Qiagen) and quantified with a Nanodrop 2000 Spectrophotometer (Agilent Technologies).

Tissue sections were deparaffinised, dehydrated and endogenous peroxidase activity was quenched with a 15 minute incubation in a hydrogen peroxide/methanol solution (30 ml H_2_O_2_ in 1000 ml methanol; VWR). Sections were labelled using a modified version of a previously published ISH protocol [21]. Briefly, sections were pre-treated with proteinase K (20 μg/ml, Roche Applied Science) for 30 minutes at 37°C and immersed in 0.3% Triton X-100 (Sigma-Aldrich). The biotin-labelled magpie reovirus and PEDV probes (500 ng/ml) were added to hybridisation buffer [100 mM Tris, 0.9 M NaCl, 0.1% sodium dodecyl sulphate (SDS) in DEPC treated water, pH adjusted to 7.2 with 1 M HCl, (VWR)], denatured at 95°C for 2 minutes, and chilled immediately on ice before application to the sections overnight at 37°C. After post-hybridisation washes, the hybridisation was amplified using an avidin-biotin-peroxidase conjugate (Vectastain ABC elite, Vector Laboratories) and visualised with 3,3′-diaminobenzidene tetrahydrochloride (Sigma-Aldrich), before the sections were counterstained with Mayer’s haematoxylin (Surgipath) and permanently mounted. ISH for ARV demonstrated labelling predominantly of mononuclear cells in areas of necrosis and surrounding parenchyma in the liver and spleen (Figure 2C and D).

### Magpie reovirus characterisation

The open reading frame (ORF)-3 region of the small 1 (S1) gene segment of the magpie reovirus genome, which codes for the sigma C protein, was amplified. Use of this variable surface protein has been recommended by previous studies for ARV strain characterisation [22,23]. The primer pairs, forward 5′-atggcgggtctcaatccat-3′ and reverse 5′-gatgcckgtacgcacggt-3′, were employed to amplify the ORF-3 using Fast Cycling PCR Kit (Qiagen) and PCR conditions of 95°C for 5 minutes, followed by 40 cycles of 95°C for 30 seconds, 55°C for 30 seconds and 72°C for 2 minutes. PCR product purification, DNA sequencing and scans of sequence reads for ambiguities were performed as described above. Deduced amino acid sequences of the magpie reovirus ORF-3 and those of other reoviruses were aligned using the MegAlign software of the DNASTAR Lasergene Core Suite (DNASTAR, Inc.). Phylogenetic analysis was undertaken using Molecular Evolutionary Genetics Analysis (MEGA) version 5.03 [24] to construct a neighbour-joining tree.

**Figure 4:**
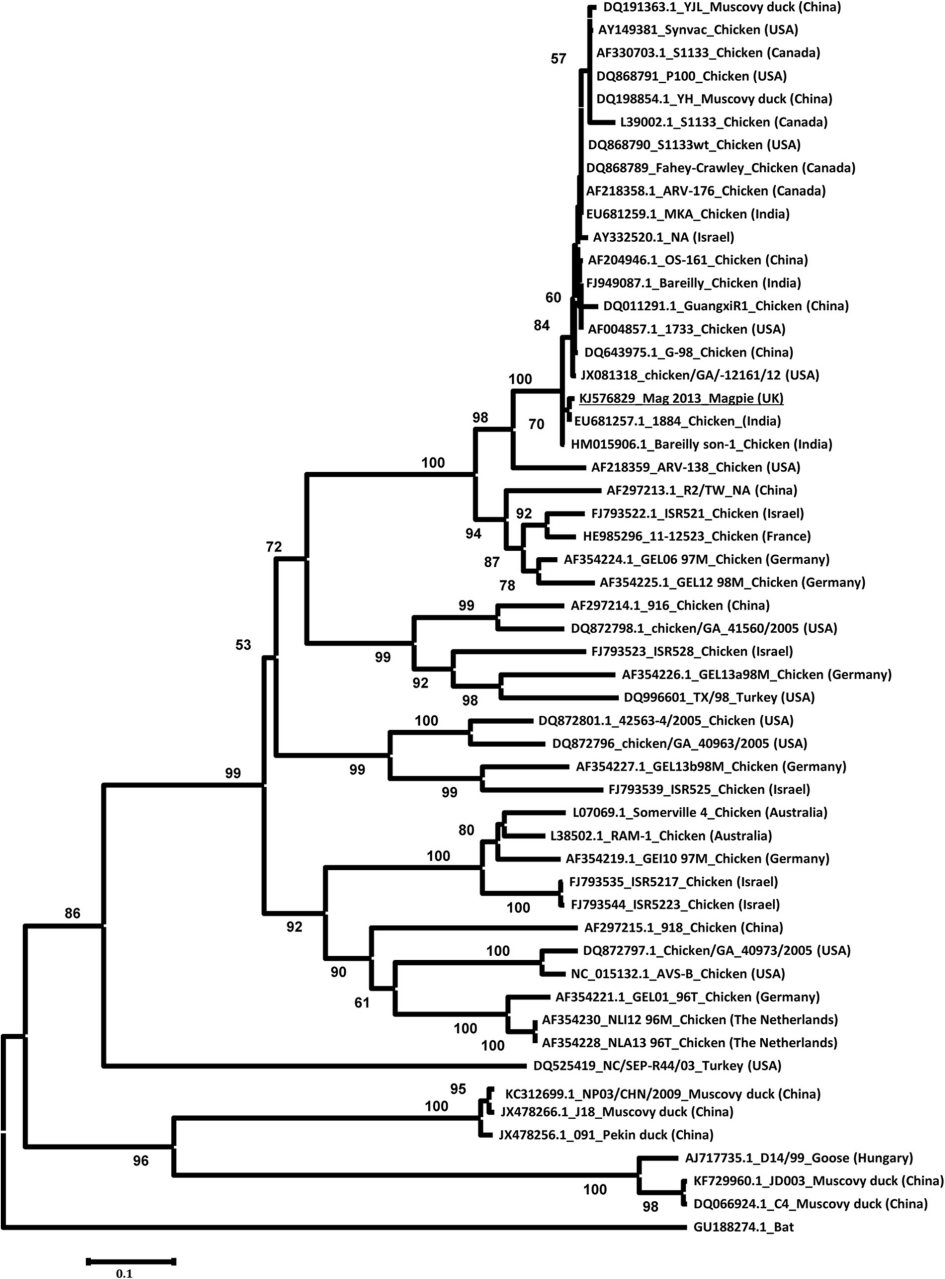
**Avian reovirus phylogeny for 285 amino acids of sigma C protein.** Phylogeny was constructed using the neighbour-joining method implemented in the MEGA 5.03 software. Each sequence on the tree is identified by GenBank accession number, isolate name, host and country of origin. The magpie reovirus is underlined and fruit bat reovirus is included as an outgroup. The reliability of phylogenetic tree branching was assessed in bootstrap analysis with 2000 replications. Bootstrap values less than 50% are not shown. NA; not available.

The neighbour-joining phylogenetic tree constructed on 285 amino acids of the magpie reovirus sigma C protein (GenBank accession number KJ576829) shows that it clustered in a well-supported clade with viruses isolated mainly from poultry, with a global distribution including India, USA and China, and distinct from representative ARVs from continental Europe (Figure 4). Amplification and direct Sanger sequencing of the sigma C protein gene from the cell pellet derived from the fourth tissue culture passage demonstrated identical sequence to that derived from the post-mortem tissue samples.

### Discussion

Here we describe a case of ARV infection in a single free-living adult magpie from southern England. This report of ARV infection associated with disease and mortality in a wild bird in Great Britain has implications for wild bird disease surveillance and our understanding of ARV transmission between domestic and wild birds. Corvids are known to be highly sensitive to West Nile virus infection in North America; therefore, they are used as a sentinel for flavivirus surveillance in the USA [25] and in Europe [14]. Corvids are numerous and frequently considered to be a pest species, but there is a relative paucity of information on their infectious diseases [26-29]. ARV infection should be included as a differential diagnosis for magpie morbidity or mortality.

Previous reports of ARV infection in free-living corvids have reported haemorrhagic and necrotising enteritis as a significant macroscopic abnormality [10,11,13]. Blood-stained SI contents and SI serosal vessel congestion were noted in this magpie. Autolysis of the SI tract precluded histopathological examination, making it impossible to further investigate the significance of these gross observations. The liver and spleen were severely affected in this magpie, similar to descriptions of ARV disease in psittacines [1]. Splenic necrosis has also been reported in cases of ARV infection in crows [11]. Neurological signs associated with reovirus infection have been reported in other corvids [12]; gross evidence of meningeal vessel congestion was seen in this case, but histopathological examination of the brain was unremarkable.

This magpie had evidence of chronic disease: it had poor body and plumage condition, dehydration and adrenocortical hyperplasia. The bird may have had generalised immunosuppression, as evidenced by splenic lymphoid atrophy and necrotising mycotic stomatitis associated with *Aspergillus fumigatus*, which often is an opportunistic pathogen. Bacterial and fungal co-infections and lymphoid necrosis have been features of ARV infections in other avian species [1,2]. Since histopathological examinations failed to identify disseminated bacterial colonies or a florid inflammatory response, recovery of *Escherichia coli* from multiple sites was considered more likely to be the result of post-mortem tissue invasion than disseminated systemic infection.

The amino acid sequence obtained from the magpie reovirus sigma C protein grouped with greatest similarity with ARVs from poultry with a widespread distribution in China, India, Israel and North America. Only a few ARV sigma C protein sequences are publicly available from North Western Europe (all from poultry, none from the UK), but they are distinct from the clade which contains the UK magpie reovirus (Figure 4). It is plausible that wild birds may act as a reservoir of infection for poultry or *vice versa*. Further interpretation, however, is currently limited since the coverage and representativeness of available ARV sequences is not systematic (in space, time or by avian species). Additional sequence data of ARVs from both poultry and wild birds is required to elucidate the dynamics and risk pathways of ARV transmission between captive and free-living birds.

## Conclusions

We describe severe hepatic and splenic necrosis in a single magpie with ARV infection. To the authors’ knowledge, this is the first report of ARV infection associated with significant disease in a wild bird in Great Britain. Continued surveillance is required to determine the frequency of magpie mortality incidents associated with magpie reovirus and the distribution and prevalence of ARV infections in other wild bird species. Further ARV sequence data is required to inform the epidemiology and transmission of ARVs between wild birds and poultry. Any implications of this magpie reovirus for poultry and other captive bird health should be investigated.

## Abbreviations

ARV: Avian reovirus
FCS: Foetal calf serum
ISH: *In situ* hybridisation
SI: Small intestinal
TEM: Transmission electron microscopy
